# Miniaturized MIMO Antenna Array with High Isolation for 5G Metal-Frame Smartphone Application

**DOI:** 10.3390/mi13071064

**Published:** 2022-07-01

**Authors:** Yuehui Gao, Junlin Wang, Xin Wang, Rui Shao

**Affiliations:** College of Electronic Information Engineering, Inner Mongolia University, Hohhot 010021, China; gaoyh0703@163.com (Y.G.); 17853313635@163.com (R.S.)

**Keywords:** multiple-input-multiple-output (MIMO) antenna, N79 band, high isolation, metal frame, SAR

## Abstract

In this paper, a highly isolated multiple-input multiple-output (MIMO) antenna array is proposed for fifth-generation (5G) metal frame smartphones. The eight identical small-sized inverted F-shaped folded slots are etched on the metal frame as a MIMO antenna. The bandwidth of the antenna can be adjusted by changing one of the short branches of the antenna. The bandwidth of the antenna can reach the N79 band (4.4~5.0 GHz). By carefully arranging the positions of the eight antenna elements, ideal spatial diversity can be successfully achieved to mitigate the coupling between the antenna elements effectively. What is more, a small combination slot of C-shape (0.0078 × 0.047λ^2^) and vertical I-shape (0.12 × 0.004λ^2^) between each antenna element is introduced to improve the element isolation of the MIMO antenna system. The proposed MIMO array has been simulated, fabricated, and measured. The results show good impedance matching (return loss > 6 dB) and high isolation (>22 dB). Due to the decent element isolation, the envelope correlation coefficient (ECC) between each antenna element is below 0.049. It can provide a reliable anti-interference performance for the MIMO antenna system. In addition, the measured radiation efficiencies of the MIMO antenna system are higher than 50%. The interaction of the hand model with the MIMO antenna system is also investigated, including the specific absorption rate (SAR).

## 1. Introduction

With the rapid development of the fifth-generation (5G) mobile communication technology, the demand for higher data transmission and low latency in communication systems is increasing. Compared to 4G communication, 5G communication has noticeable improvements in the throughput, channel capacity, delay, and other aspects [1]. To achieve these goals, many academic researchers and even application-oriented industry people have begun to focus on multiple-input multiple-output (MIMO) technology [2]. However, due to the limited space in smartphones, it will face tough challenges in implementing 5G MIMO antenna technology while achieving high isolation between antennas and wide operating frequency bands.

In recent years, various types of MIMO antennas for 5G smartphones have been reported [3,4,5,6,7,8,9,10,11,12,13,14,15,16,17,18,19,20,21,22,23,24,25,26,27,28,29,30,31,32,33,34,35,36], which fully demonstrate their superiority in the channel capacity, transmission rate, and throughput. Some of these reported can cover a single-frequency narrowband band (about 200 MHz) in the Sub-6 GHz spectrum [3,4,5,6,7,8,9,10,11,12,13,14,15,16,17,18]. The other designs have shifted to the dual-band, tri-band, wideband, and ultra-wideband to improve throughput [19,20,21,22,23,24,25,26,27,28,29,30,31,32,33,34,35,36]. These MIMO antenna systems in smartphones with metal frames have been proposed among [14,17,25,30,34,35,36]. However, their bandwidths are limited due to the harsh electromagnetic environment brought by the metal-frame design in [14,17,25]. The MIMO antenna systems in [30,34,35,36] significantly improve this problem. Ref. [30] introduces a wideband antenna with slots on the metal frame and back cover, but the antenna uses lumped elements to adjust impedance matching and uses the differential balun chips for differential feeding, which increases the complexity of the antenna design. This is not the target that we pursue. In [34,36], the designed broadband antennas are very similar. The main contribution to the broadband is the slotted part of the back cover. It is at the expense of a larger size, which is also far higher than the goal we pursue. In the designs of [31,32,33], the MIMO antenna system is integrated into the metal back cover of the mobile phone. However, the distance between the radiation patch and the ground is too large to integrate into practical application. If the metal back covers with a large area is used as the antenna ground, it will affect the radiation performance making the impedance broadband not reach the –6 dB requirement [31].

Various types of decoupling techniques reported are effective methods to raise the isolation between each MIMO antenna element, such as parasitic decoupling techniques [3], neutral lines [4,6], and loading decoupling elements [16]. It is reported in [5,37,38] that the diversity technique can increase the space between the antennas to improve isolation. Both frequency selective surfaces [39] and metasurfaces [40] have been used to mitigate mutual coupling in MIMO antenna systems. Additionally, I-shaped and H-shaped ground slots are introduced between the antenna elements to improve the element isolation in [4,35], respectively. In [41], the small T-shaped open slot is used to reduce the coupling between two closely spaced antenna elements, too. However, a chip capacitor is loaded to reduce the size of the slot in this method (parasitic scatterers decoupling). The isolation can be adjusted by changing the capacitor value, but this method is limited in reducing the size of the isolation element. Plus, the isolation level is restricted by two aspects (T-shaped slot size and the chip capacitor value), which increases the complexity of the antenna. Inspired by [35,41], we presented C-shaped and I-shaped combination slots between the antenna elements. The footprint can be reduced to 3 mm × 0.5 mm (0.0078 × 0.047λ^2^ (λ corresponds to 4.7 GHz)). Moreover, spurred to [5], the spatial diversity isolation is adopted to arrange the positions of the antennas carefully. By mixing the two isolation methods, the number of ground slots can be reduced to four.

In the latest reported works [42,43,44], MIMO antennas are all designed with microstrip patches. The back cover of the design [42] has clearance areas which is not all metal. In the design of [43,44], the size of the antenna is large to satisfy the wide bandwidth. The planar structure is adopted in [45,46] to integrate the antenna with the back cover of the mobile phone, but the bandwidth of the designs is also affected by the metal back cover. In [45], the antenna obtains a narrow bandwidth since the footprint of the metal back cover is larger than the antenna itself. The methodology in [46] does not use a large metal ground, so it is not conducive to integration in the metal back cover. The designs in [42,43,44,45,46] are not suitable for 5G metal bodies but for non-metal bodies.

This paper focuses on MIMO antenna systems for 5G metal frame smartphone applications and addresses the common problems of MIMO antenna systems discussed above, such as large size, complex structure, and low isolation. We etched eight inverted-F-shaped grooves as an 8 × 8 MIMO antenna system for the metal bezel of a 5G smartphone. The proposed MIMO antenna system can cover the entire N79 5G frequency band (4.4~5.0 GHz). Facing the restricted radiation environment brought by the metal frame, we have explored some radiation methods for MIMO antennas in such case. The small slot is etched as an antenna on the metal frame. What is more, the position of each antenna is carefully arranged so that it can be beautiful and leave redundant space for the design of 2G/3G/4G antennas. More importantly, the small-size combined slot with the C-shape and vertical I-shape connected to each other is proposed to improve isolation. The isolation in the target band can be achieved at 22 dB, and ECC is less than 0.049. Ideally, the total efficiency is 55% to 78%. Simulation and measurement results of the 8 × 8 MIMO antenna show satisfactory performance. In addition, the effect of the human hand model and the impact of the antenna on the user have also been researched. It shows great potential in the 5G mobile metal frame terminal application.

## 2. Proposed MIMO Antenna Design

**Figure 1:**
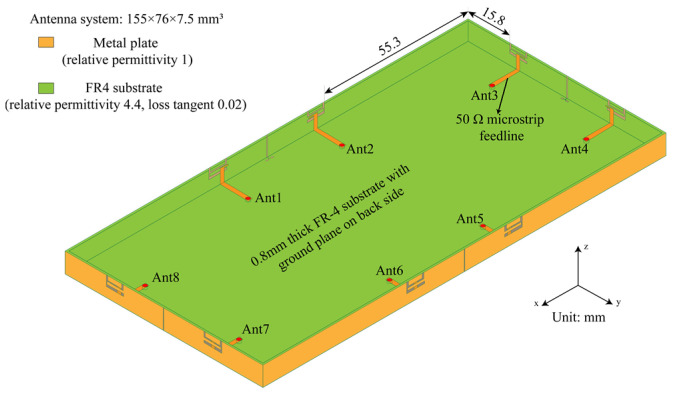
Overall geometry and dimensions of the proposed MIMO antenna array.

Figure 1 shows the geometry of the proposed MIMO antenna system with the metal frame for the 5G smartphone. The ground is 155 × 76 mm^2^ and the thickness of the smartphone is 7.5 mm. The distances between the etched antenna slot on the long bezels and the corners of the entire body edge are 55.3 mm. The distances between the etched antenna slot on the short bezels and the corners of the entire body edge are 15.8 mm. Obviously, there is still design room for 2G/3G/4G terminal antennas and other equipment jacks. In this design, an FR-4 substrate (relative permittivity 4.4, loss tangent 0.02) with a thickness of 0.8 mm is used for the body construction of the smartphone. At the same time, we used copper (finite conductivity) with very stable conductivity (conductivity 5.8 × 10^7^ S/m) and little effect on impedance matching as the radiator. The simulation results of the proposed antenna system were obtained by HFSS Software. During HFSS calculation, the adaptive meshing was performed on a single frequency point (4.75 GHz). After the meshing was completed, the solutions of other frequency points under the same solution setting item were based on the previous setting. The meshing was performed at the fixed frequency point (the maximum number of passes was 20). Radiation boundaries in all directions were more than λ_0_/4 far from the radiator (λ_0_ corresponds to a frequency of 4.75 GHz).

### 2.1. Antenna Element Design

**Figure 2:**
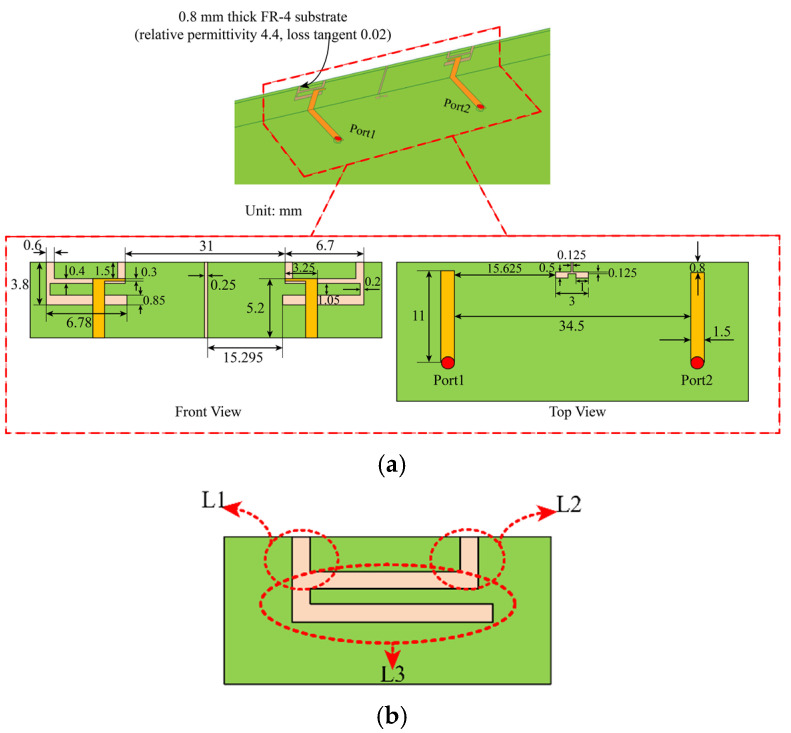
Detailed dimensions of the antenna element. (**a**): Inverted L-shaped microstrip line and de-coupling element and (**b**): no optimized antenna element.

**Figure 3:**
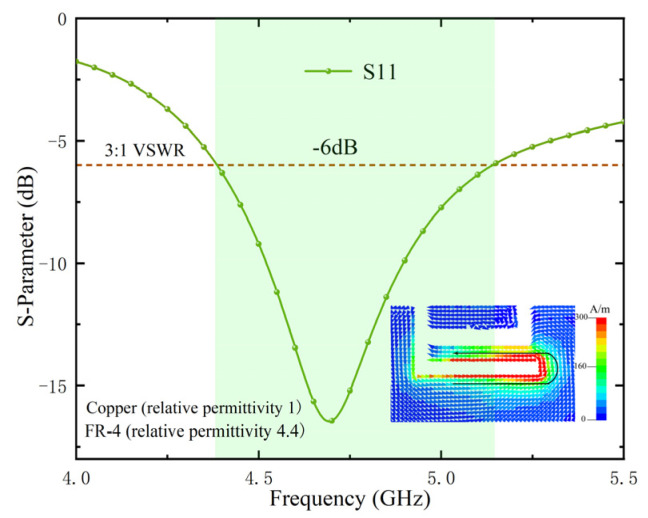
Surface current distributions of the resonance and simulates the reflection coefficient of the antenna element.

The planar structure and detailed dimensions of the proposed adjacent antenna elements are shown in Figure 2a. Inverted-F antennas (IFAS) are widely used in terminal antennas due to their small size. Based on the principle of transmission line, we have assumed that the entire length of L3 is close to the λ/4 electrical length, which excites resonance and produces an impedance bandwidth. L3 is shown in Figure 2b. To verify this hypothesis, we tried to etch the inverted F-shaped folded groove on the metal frame and used the inverted L-shaped microstrip line for feeding to transfer the energy into the inverted-F groove by coupling. Plus, the resonance was successfully excited to form the impedance bandwidth required. The resonant surface currents and the reflection coefficients of the simulated antenna elements are shown in Figure 3. It is clear that the resonant surface current is mainly distributed along with the L3 slot of the IFA slot. This result is consistent with the hypothesis.

**Figure 4:**
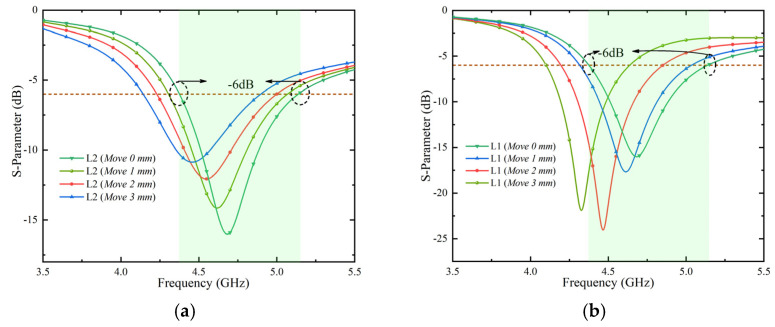
Simulate reflection coefficient of the antenna element. (**a**): With effects of L2 distance moved and (**b**): with effects of L1 distance moved.

In addition, the effect of the distance between the two short slots (L1 and L2) on the impedance bandwidth has been studied, as shown in Figure 2b. The result in Figure 4a shows that moving L2 to the right will lower the frequency and reduce the impedance matching. Figure 4b shows that moving L1 to the left also lowers the frequency. The two simulation results show that the resonance length of L3 can be changed by adjusting the distance of L1 and L2, and that the resonance frequency can be shifted. By comparing the simulation results, the effect is the best when the distance between L1 and L2 is shown in Figure 2a front view. The target of our proposed IFAS is to cover the 4.4~5.0 GHz operating bandwidth.

**Figure 5:**
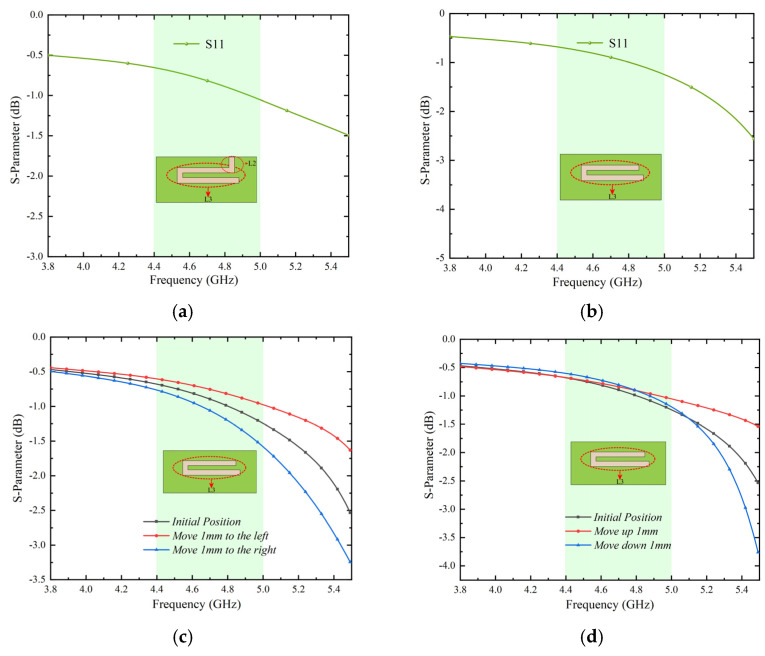
Simulation results. (**a**): Reflection coefficients without L1, (**b**): reflection coefficients without L1 and L2, (**c**): the reflection coefficients obtained after moving the feeding line to the left and right, respectively, and (**d**): the reflection coefficient obtained after moving the feeding line up and down, respectively.

Next, let us discuss why L1 and L2 slots are needed for the antenna. Figure 5 shows the simulation results of reflection coefficients in four cases. The simulation results for the reflection coefficient of the antennas with L1 and without L1 and L2 are shown in Figure 5a,b. It is obvious from two results that resonance cannot be achieved in the desired frequency band in either situation. L1 and L2 are thus needed. When the relative position between the feeding line and the L3 is changed, the simulation results for the reflection coefficient of the antenna without L1and L2 are shown in Figure 5c,d. These two findings demonstrate that resonance in the band cannot be attained. If there is simply L3, L3’s length needs to be increased in order to achieve resonance in the band. This is not what we had hoped for.

**Figure 6:**
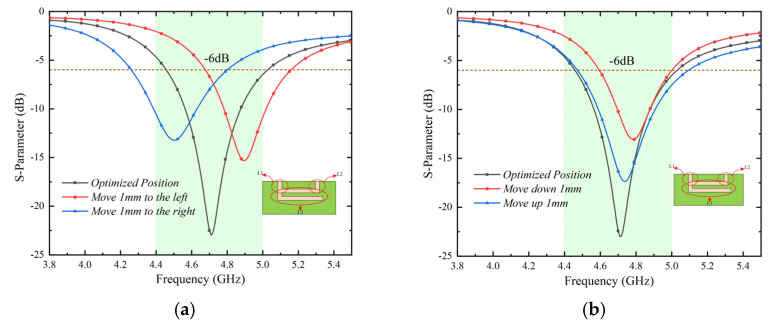
Simulation results with L1 and L2. (**a**): The reflection coefficients obtained after moving the feeding line to the left and right, respectively, and (**b**): the reflection coefficients obtained after moving the feeding line up and down, respectively.

Figure 6 shows the simulated results for the reflection coefficient of a single antenna when the relative position between the feeding line and L3 is adjusted. The simulation findings show that the relative position between the feeding line and L3 has an impact on the impedance matching to some extent. The optimized location has the best matching effect.

**Figure 7:**
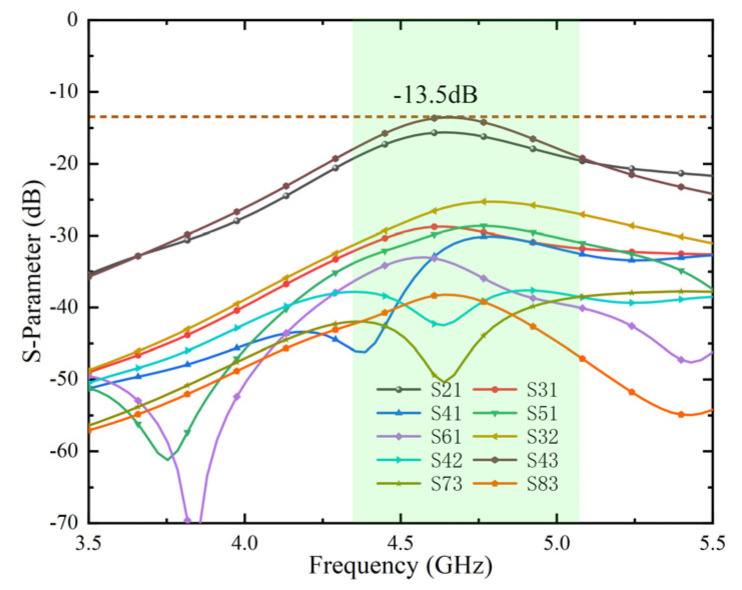
Transmission coefficients of MIMO antenna systems without decoupling.

The isolation of the arranged MIMO antenna system without any decoupling structure can reach more than 13.5 dB, as shown in Figure 7. The MIMO antenna systems are fully capable of terminal products with low isolation requirements and limited clearance. A decoupling structure with a tiny footprint for terminal products with high isolation requirements was introduced to make the adjacent antenna elements (Ant 1, Ant 2 and Ant 3, Ant 4 and Ant 5, Ant 6 and Ant 7, and Ant 8) obtain higher isolation.

### 2.2. Decoupling Element

We have experimented with several different decoupling techniques to improve isolation between each two antenna elements. Due to their inability to control the surface current on the metal frame, we find that few solutions work well with our design. In our design, the metal frame’s surface current plays a major role in the coupling between two antenna elements.

**Figure 8:**
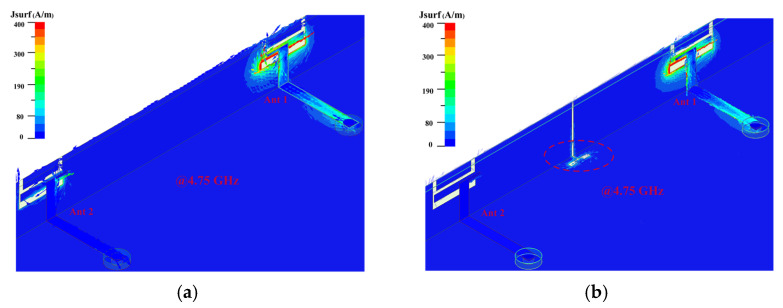
Surface current distributions when Ant 1 is excited. (**a**): Without decoupling structure and (**b**): with decoupling structure.

We separated the two antenna elements with a slot etched on the metal frame. At the same time, a groove is etched on the ground to cancel the coupled currents. In Figure 8a,b, the decoupling effect is clearly visible. Miniaturization is accomplished by making the decoupling slot’s footprint as small as possible. The decoupling structures’ ultimate size and shape have both been optimized, which is noteworthy.

A C-shape slot with an area of only 0.0078 × 0.047λ^2^ (λ corresponds to 4.7 GHz frequency) is etched on the back cover of the metal frame. A vertical I-shape with an area of 0.12 × 0.004λ^2^ slot is engraved on the metal frame. The two grooves are connected with each other. The etched combination groove can block and confine the current on the upper surface of the metal frame, which can prevent the excited antenna’s field from reaching the unexcited area.

**Figure 9:**
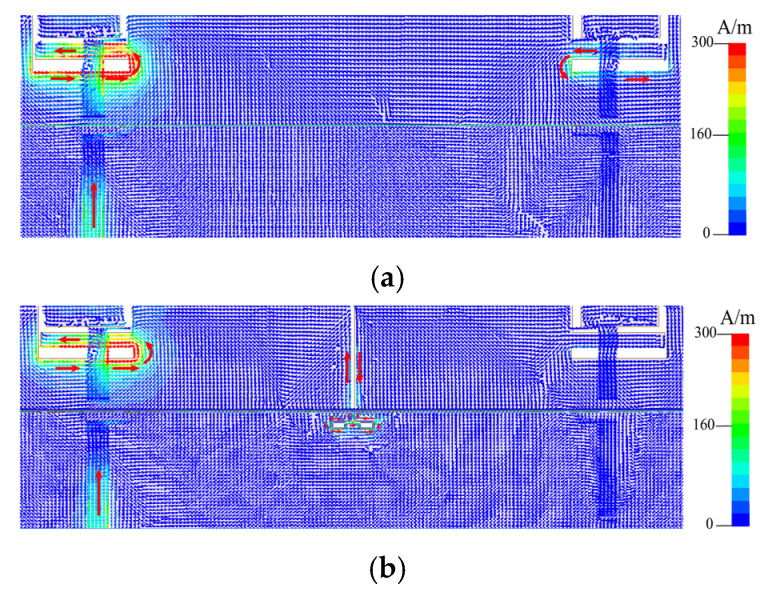
Surface vector current distributions. (**a**): Without decoupling structure and (**b**): with decoupling structure of Ant 1 and 2.

**Figure 10:**
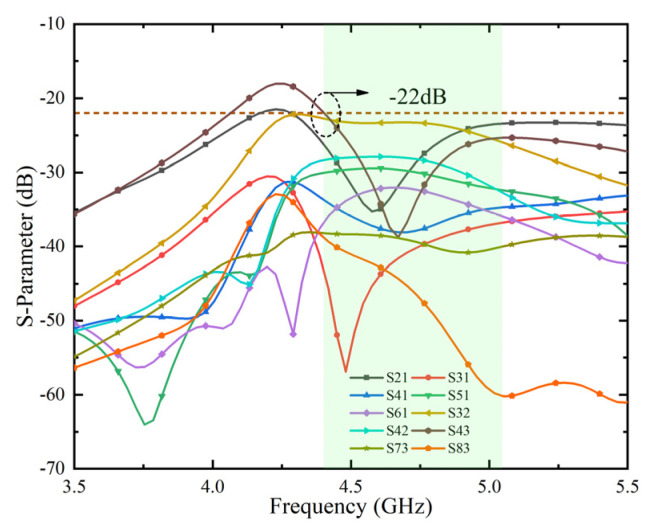
Transmission coefficients of MIMO antenna systems with decoupling.

The surface vector current distribution without the decoupling slot and with the decoupling slot is compared, as shown in Figure 9, to verify the mechanism of the decoupling slot. According to Figure 9a,b, it is concluded that the combination decoupling slot effectively blocks the current excited by Ant 1 from coupling into Ant 2. By comparing Figure 7 and Figure 10, it is evident that adding a decoupling slot improves the isolation from 13.5 dB to more than 22 dB, which is comparable to the latest technology in this field. Finally, four combination decoupling slots have been etched entirely to improve the isolation of four pairs of adjacent antenna elements. The size of the entire MIMO antenna system has been optimized. The optimized results are shown in Figure 2a.

## 3. Results and Discussion

**Figure 11:**
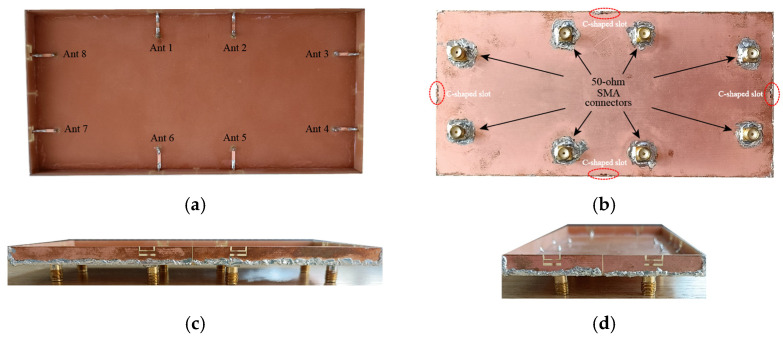
Photographs of the fabricated MIMO antenna system. (**a**): Front view, (**b**): back view, (**c**): long side view, and (**d**): short side view.

**Figure 12:**
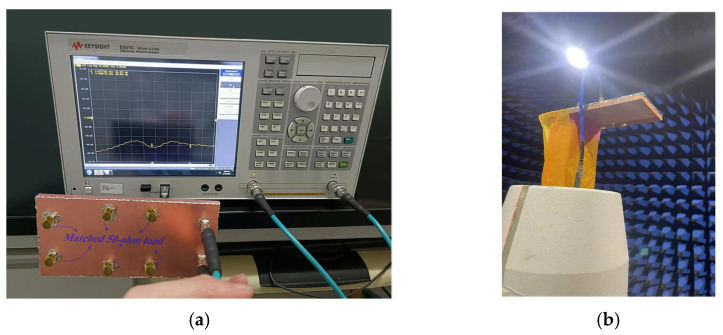
Photographs of the test site where the MIMO antenna was tested. (**a**): Vector network measurement analyzer and (**b**): SATIMO anechoic chamber.

A prototype of the MIMO antenna system was manufactured and tested, as shown in Figure 11, to verify the feasibility of the proposed 8-element MIMO antenna system. Metallic copper was attached to the bottom and side panels of the smartphone’s body made of RF-4. Microstrip feeders were printed on the top, as shown in Figure 11a. Moreover, the metal frame and the ground plane of the mainboard were welded together. Eight 50 Ω SMA connectors were soldered with the feeder for convenient antenna testing. The red circled part shown in Figure 11b is the etched C-shape decoupling slot. As shown in Figure 11c,d, the gap between the periphery of the frame and the bottom plate is welded to complete by solder. The S-parameter test results were obtained by the Agilent E5071C vector network measurement analyzer, as shown in Figure 12a. The far-field results were obtained by a SATIMO anechoic chamber, as shown in Figure 12b.

### 3.1. S-Parameters and Efficiency

Thanks to the symmetrical arrangement of the MIMO antenna system, only the simulation and measurement results of the part of the antenna elements are analyzed and compared here.

**Figure 13:**
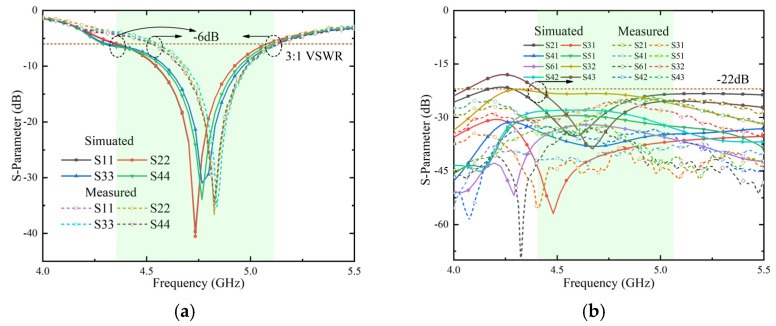
Simulated and measured. (**a**): Reflection coefficients and (**b**): transmission coefficients of MIMO antenna system.

The simulated and measured S-parameter (reflection coefficient) results are given in Figure 13a. Due to manufacturing tolerances, the measured bandwidth is slightly offset, but the overall measurement results are satisfying. The measured impedance bandwidth can reach 12.7% within the acceptable range and the center frequency shifted to 4.82 GHz. The transmission coefficients between some ports (1 and 2, 1 and 3, 1 and 4, 1 and 5, 1 and 6, 2 and 3, 2 and 4, and 3 and 4) with relatively strong coupling are shown in Figure 13b. It is worth noting that the isolation degree is 2 dB higher than the simulation result because it is very difficult to weld the metal frame and the ground plane of the mainboard when the MIMO antenna system is manufactured, which is some gap. Overall, the isolation level between components exceeds 22 dB in the simulated and measured frequency bands.

**Figure 14:**
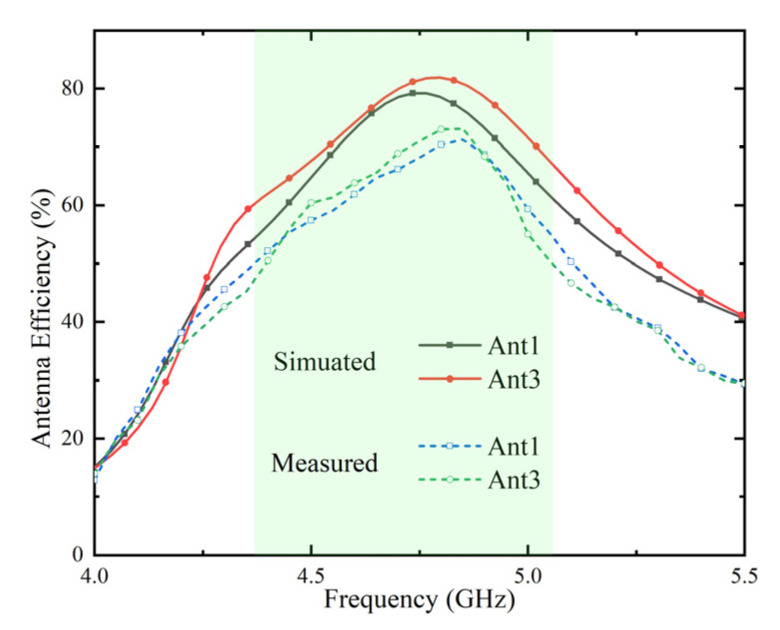
Simulated and measured antenna efficiencies.

In Figure 14, the measured radiation efficiency of Ant 1 and Ant 3 is lower than the simulated result in the frequency band, and the peak of the center frequency is also slightly lower than the simulated value. This is due to the fact that during the test, in addition to manufacturing tolerances, the placement of the antenna system can also cause the loss of tangent of the FR-4 substrate to be non-uniform. However overall, the measured efficiencies of the Ant 1 and Ant 3 range from 50~73% (−3 dB~−1.37 dB) in the desired frequency band.

### 3.2. MIMO Performances

Therefore, some antenna elements (Ant 1 and Ant 2, Ant 1 and Ant 3, Ant 1 and Ant 4, Ant 1 and Ant 5, Ant 1 and Ant 6, Ant 2 and Ant 3, Ant 2 and Ant 4, and Ant 3 and Ant 4) with similar positions and strong radiation interference, were simulated and tested. The ECC values between these antenna elements can be calculated according to the calculation Equation (1) [24]:

(1) $$\rho_{e}=\frac{{\left|\iint_{4\pi }F_{1}^{\to^{*}}\left(\theta ,\phi \right)\cdot F_{2}^{\to^{*}}\left(\theta ,\phi \right)d\Omega \right|}^{2}}{\iint_{4\pi }{\left|F_{1}^{\to^{*}}\left(\theta ,\phi \right)\right|}^{2}\cdot d\Omega \times {\iint_{4\pi }|F_{2}^{\to^{*}}\left(\theta ,\phi \right)|}^{2}d\Omega }$$

where *F_i_*→*(0, φ) denotes the *i*th antenna’s complex three-dimensional (3-D) radiated far-field pattern.

**Figure 15:**
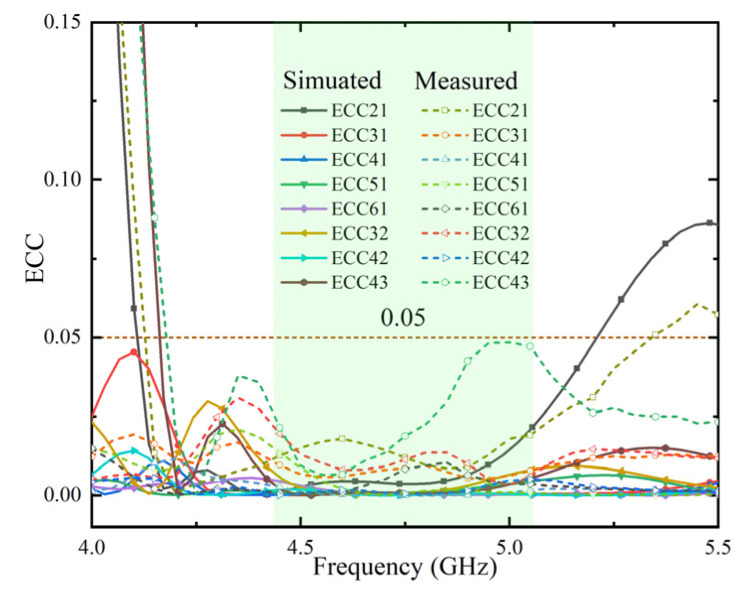
Simulated and measured of ECCs between some near antenna-element.

The results are shown in Figure 15. Clearly, the level of ECC is below 0.049 in the desired frequency band, which provides promising MIMO diversity performance.

### 3.3. Radiation Performances

**Figure 16:**
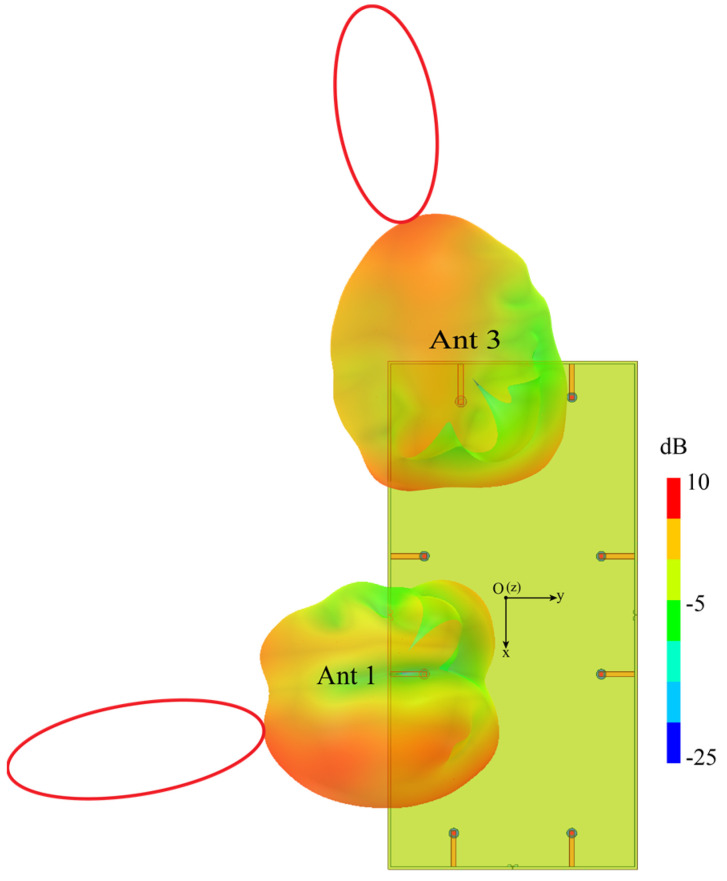
Simulated 3-D radiation patterns at 4.75 GHz.

In this subsection, the radiation patterns of MIMO antenna systems were studied. Since the system is symmetrically arranged, for simplicity, only the 3-D radiation patterns of Ant 1 and Ant 3 were analyzed. The simulation results at 4.75 GHz are shown in Figure 16. Obviously, in the XY plane, the radiation patterns of Ant 1 and Ant 3 point to the negative *y*-axis direction and the negative *x*-axis direction, respectively, showing orthogonal characteristics. The maximum radiation directions do not point to each other, which is beneficial to spatial diversity. The ECC value below 0.049 is also obtained, while the isolation is improved.

**Figure 17:**
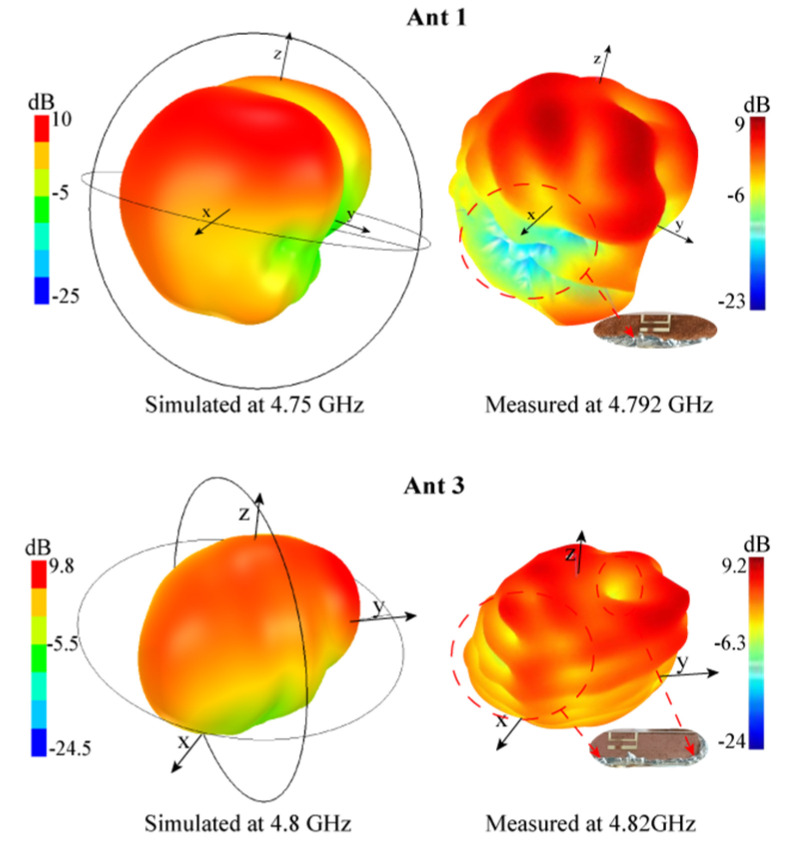
Simulated and measured 3-D radiation patterns of Ant 1.

The simulated and measured 3-D radiation patterns of Ant 1 at 4.75 GHz and Ant 3 at 4.8 GHz are depicted in Figure 17, respectively. Obviously, the measured 3-D radiation pattern surfaces of Ant 1 and Ant 3 at the frequency points are not smooth. The reason is that the surface of the welding place is uneven to generate electrostatic effects, as shown in Figure 17 (the area pointed by the red dotted arrow). However, overall, Figure 17 shows the measured 3D radiation patterns agree well with the simulated ones.

### 3.4. User’s Hand Effects and SAR Analysis

In this subsection, the impact of user hand models was studied. It is worth noting that the MIMO antenna system for Sub-6 GHz is only used for data transmission, so the study of the head model is not included here [5].

**Figure 18:**
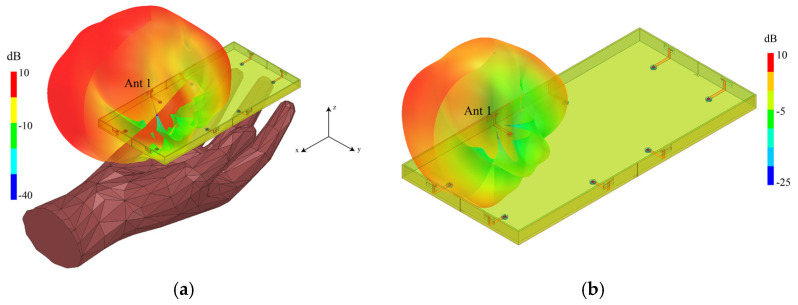
Simulated 3-D radiation patterns of Ant 1 of the proposed MIMO antenna system at 4.75 GHz. (**a**): Radiation pattern with a right-hand model and (**b**): radiation pattern without a right-hand model.

In Figure 18, the proposed MIMO antenna system is simulated with and without the user’s right-hand model. By comparing Figure 18a,b, it can be observed that human tissue forms a reflective surface so that the radiation of the entire antenna system is the largest in the direction away from the human body, while the radiation is relatively more minor in the direction close to the human body.

**Figure 19:**
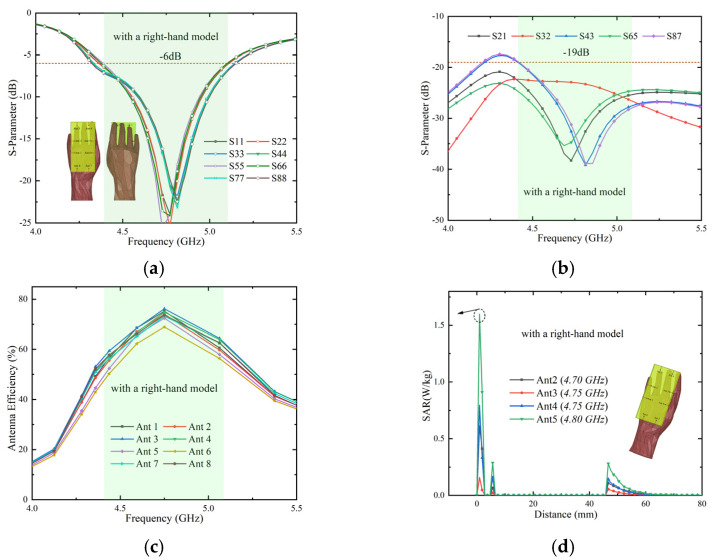
Simulation results of the proposed MIMO antenna with a right-hand model. (**a**): Reflection coefficients with a right-hand model, (**b**): transmission coefficients with a right-hand model, (**c**): total efficiency with a hand model, and (**d**): SAR with a right-hand model.

First, the designed antenna grabbed by a right hand is set up and simulated. Due to the direct contact of the fingers with the antenna, inevitable shocks may be caused to the antenna elements, resulting in some performance degradation. In Figure 19a, the impedance matching of the reflection parameter is significantly reduced. However, the effect is relatively small in the target frequency band. Figure 19b shows that the isolation drops by about 3 dB and can remain above 19 dB, which is still relatively higher in related fields. As shown in Figure 19c, the efficiency of the eight antenna elements changes only slightly and the efficiency range remains above 50%. In general, the effect of user hand shape on antenna performance is acceptable within the target frequency band.

Finally, we studied and analyzed the specific absorption rate (SAR) value. The SAR value characterizes the power absorbed from the mobile terminal to the human body. As the number of antennas increases, it remains challenging to obtain lower peak levels. Figure 19d presents the simulation results of the four antenna elements (Ant 2, Ant 3, Ant 4, and Ant 5) closer to the user’s fingers in the MIMO antenna system. It can be observed that the SAR level of Ant 2 at 4.7 GHz, Ant 3 at 4.75 GHz, Ant 4 at 4.75 GHz, and Ant 5 at 4.8 GHz are below 1.6 W/kg, which conforms to the standards formulated by the United States, Europe, and China.

### 3.5. Comparison and Discussion

To further demonstrate that the proposed work is better in design and performance, a detailed comparison of this work with the available work is conducted and illustrated in Table 1. It is worth mentioning that based on different analyses, investigations, and studies, we believe that the proposed model has the potential to be a useful MIMO system for future 5G smart mobile terminals.

First, all bandwidths are –6 dB impedance bandwidth, which is carefully extracted from these articles. The data that is superior to the proposed MIMO antenna is painted in green color. The proposed antenna is with unique features at least from one aspect: isolation, ECC, or size.

Compared with all the listed projects, the proposed antenna design has low complexity and is convenient for future practical applications. Although the proposed MIMO antennas are worse bandwidth than the reference antennas in [20,21,22,44], and worse isolation and ECC than the latest literature [42], these references are with a larger size or even with a greater design complexity simultaneously. The antenna distance of different works is also added in Table 1. The proposed design is not the antenna pair and uses spatial diversity to improve isolation, so the antenna distance is larger than the other works’.

In a word, the proposed MIMO antenna makes a good compromise between bandwidth, isolation, and size and exhibits better performance than most of the MIMO antennas reported in Table 1.

## 4. Conclusions

In the paper, the 8 × 8 MIMO antenna system with high isolation and small size (0.0595 × 0.106λ^2^) is presented for 5G metal frame smartphone applications. The designed MIMO antenna system has an ideal antenna efficiency of more than 55% in the N79 Band frequency band. The isolation below −22 dB and typical ECC (<0.049) can be achieved by two methods of the spatial diversity and the combined slot. Furthermore, the influence of the user’s hand on the antenna and the radiation effect on the user’s hand is further considered. Plus, the low peak SAR is obtained, which meets the US standard of 1.6 W/kg, and the Chinese and European standards of 2.0 W/kg. Compared to other reported MIMO antennas, the presented antenna has smaller size and leaves more design space for other antennas and components. To validate the simulation results, the prototype was made and measured. What is more, it is found that the simulated results for various key performance parameters are in very good agreement with the measured results. Based on the performance and the measured results, we believe this structure still holds great promise in the next-generation metal frame mobile terminals. Meanwhile, the proposed MIMO antenna has good scalability, and it can be designed in combination with other methods, such as introducing the higher-order modes by adding shorting pins to generate resonances.

## Figures and Tables

**Table 1:** Performance comparison of the 5G MIMO terminal antennas.

Reference	Antenna Pair	Decoupling Method	Working Band (GHz)	Isolation (dB)	Efficiency (%)	ECC	Antenna Size (mm^2^)	Complexity
[4]	×	NL and GS	3.4–3.6	>15	45–60	<0.15	10 × 6	Simple
[5]	×	BME, PoD, and PaD	3.4–3.6	>17.5	62–76	<0.05	19.5 × 3	Simple
[7]	×	PE	3.4–3.6	>19.1	59–68	<0.0125	17.4 × 6	Medium
[15]	√	PE	3.4–3.6	>17	58–75	<0.1	9.4 × 6.2	Medium
[17]	√	OP	3.4–3.6	>12.7	35.2–64.7	<0.13	25 × 1.5	Complex
[18]	√	OP	3.4–3.6	>20	48.6–54.5	<0.02	15 × 7	Complex
[20]	√	Without	LB: 3.4–3.6 HB: 4.8–4.9	>11.8	43–52-	<0.2	15 × 4.5	Complex
[21]	×	GS and SV	LB: 3.4–3.6 HB: 4.8–4.9	>8.5	38.3–39.5	<0.42	18.8^2^×π×¼	Medium
[22]	×	Without	LB: 3.4–3.6 HB: 5.51–5.93	>11.2	51–80	<0.08	15.2 × 9.6	Simple
[26]	×	SV	4.4–5.0	>22	40–50	<0.068	15.6^2^ × 2	Complex
[42]	√	CCD	3.42–3.69	>24	53–70	<0.01	16 × 6	Complex
[44]	×	Without	LB: 3.1–3.6 HB: 4.4–6.1	>18.5	50–75	<0.1	18 × 4.5	Simple
[46]	×	OCM & CCFS	3.3–3.8	>20	40–68	<0.13	27^2^ × ½	Medium
**Proposed**	**×**	**SD and GS**	**4.4–5.0**	**>22**	**50–73**	**<0.049**	**6.78** × **3.8**	**Simple**

Abbreviations: PE = parasitic element, NG = not given, NL = neutralization line, GS = ground slot, BME = balanced mode excitation, PoD = polarization diversity, PaD = pattern diversity, OP = orthogonal polarization, SV = shorted vias, SD = spatial diversity, OCM & CCFS = orthogonal characteristic modes and capacitive coupling feeding structure, CCD = chip capacitive decoupler, and SCR = shared compact radiator.

## Data Availability

We choose to exclude this statement.
